# Bladder Urothelial Carcinoma Diagnosed by Perineal Cutaneous Biopsy: A Case Report

**DOI:** 10.7759/cureus.116445

**Published:** 2026-09-17

**Authors:** Ramon J Guiu, Guilherme C Dias, Pedro H Silva, Murilo A Ferreira, Guilherme R Ventura

**Affiliations:** 1 General Surgery, Faculdade de Medicina de São José do Rio Preto (FAMERP)/Hospital de Base de São José do Rio Preto, São José do Rio Preto, BRA

**Keywords:** bladder cancer, cutaneous metastasis, perineal biopsy, urothelial carcinoma, vi-rads

## Abstract

Bladder cancer is the most common malignancy of the urinary tract, and urothelial carcinoma represents its most frequent histological subtype in Brazil. Diagnosis is conventionally established by direct biopsy of the urothelial mucosa obtained through cystoscopy with transurethral resection of the bladder tumor, which also allows sampling of the detrusor muscle for adequate staging. We report the case of a 71-year-old hypertensive man without a smoking history who initially presented with perineal induration, difficulty in defecation, and intermittent diarrhea, and was first investigated for suspected inflammatory bowel disease. After an inconclusive colonoscopy, pelvic magnetic resonance imaging demonstrated bladder wall thickening with a right inferoposterior lesion measuring up to 3.7 cm, of probable neoplastic origin, extending toward the ureteral meatus, with bilateral upstream ureteropyelocaliceal dilation and contiguity with the prostate, corresponding to Vesical Imaging-Reporting and Data System (VI-RADS) category 5. On flexible ureteroscopy, the bladder lesion was directly visualized as a necrotic area consistent with tumor; transurethral resection could not be completed because perineal induration prevented advancement of the rigid instrument. Biopsies of the bladder and urethral mucosa were obtained with the flexible ureteroscope, together with an open incisional biopsy of the perineal skin. Histopathology of the bladder and urethral specimens showed only nonspecific chronic cystitis and urethritis, whereas the perineal skin specimen confirmed invasive urothelial carcinoma, with immunohistochemical positivity for GATA-binding protein 3 (GATA3) and cytokeratin 7 (CK7). The patient was later readmitted with postrenal acute kidney injury due to tumor invasion of the ureteral meatus, progressing to severe obstructive uropathy, and the disease had a fatal outcome despite bilateral nephrostomy and antibiotic therapy. This case illustrates cutaneous perineal biopsy as an unconventional but decisive diagnostic route when standard transurethral sampling is unfeasible or inconclusive.

## Introduction

Bladder neoplasms represent the most common malignant tumor of the urinary system, and urothelial carcinoma is their most frequent histological subtype in Brazil. According to the Brazilian National Cancer Institute (INCA), an estimated 11,370 new cases and 4,585 deaths were projected for 2020 [1]; more recent INCA estimates for the 2023-2025 triennium project approximately 7,870 new cases per year among men and 3,500 among women, confirming the ongoing relevance of this disease [2]. Among men, it ranks among the most prevalent cancers, being particularly frequent in the more developed southeastern region of the country.

Clinical suspicion typically arises in the setting of microscopic or macroscopic hematuria, which may be accompanied by irritative symptoms such as nocturia, dysuria, and urgency, particularly in patients 40 years of age or older who have been exposed to risk factors. Although tobacco use is the main risk factor, other well-established risk factors include occupational and environmental exposure to aromatic amines and industrial chemicals (as in the dye, rubber, leather, and painting industries) and a family history of the disease [3,4].

A positive urinary cytology may raise clinical suspicion, but the diagnosis is confirmed only by histopathological examination, usually obtained through cystoscopy with transurethral resection of the bladder tumor, with the goal of including the detrusor muscle layer to allow adequate staging. Situations in which conventional transurethral sampling fails to establish the diagnosis are uncommon and may require alternative diagnostic strategies, as illustrated by the case reported here.

## Case presentation

A 71-year-old man with a history of hypertension and no history of smoking initially sought medical care for perineal induration accompanied by difficulty in defecation and intermittent episodes of diarrhea. He was first evaluated by the colorectal surgery team, and the initial clinical suspicion favored an inflammatory bowel disease. A colonoscopy was performed but yielded inconclusive findings, and the diagnosis remained uncertain. On initial digital rectal examination, no palpable prostatic nodule was identified; the main clinical finding was the perineal induration.

Given the diagnostic uncertainty and the persistence of perineal induration, contrast-enhanced magnetic resonance imaging (MRI) of the abdomen and pelvis was obtained. It demonstrated diffuse thickening of the bladder wall, with a right inferoposterior lesion measuring up to 3.7 cm, of probable neoplastic origin, extending toward the ureteral meatus bilaterally, with associated upstream ureteropyelocaliceal dilation and contiguity with the prostate. The lesion showed restricted diffusion with a corresponding low signal on the apparent diffusion coefficient map, and the overall findings corresponded to Vesical Imaging-Reporting and Data System (VI-RADS) category 5 (Figure 1) [5]. These findings redirected the investigation toward a primary bladder lesion, and the patient was referred to the urology department.

**Figure 1:**
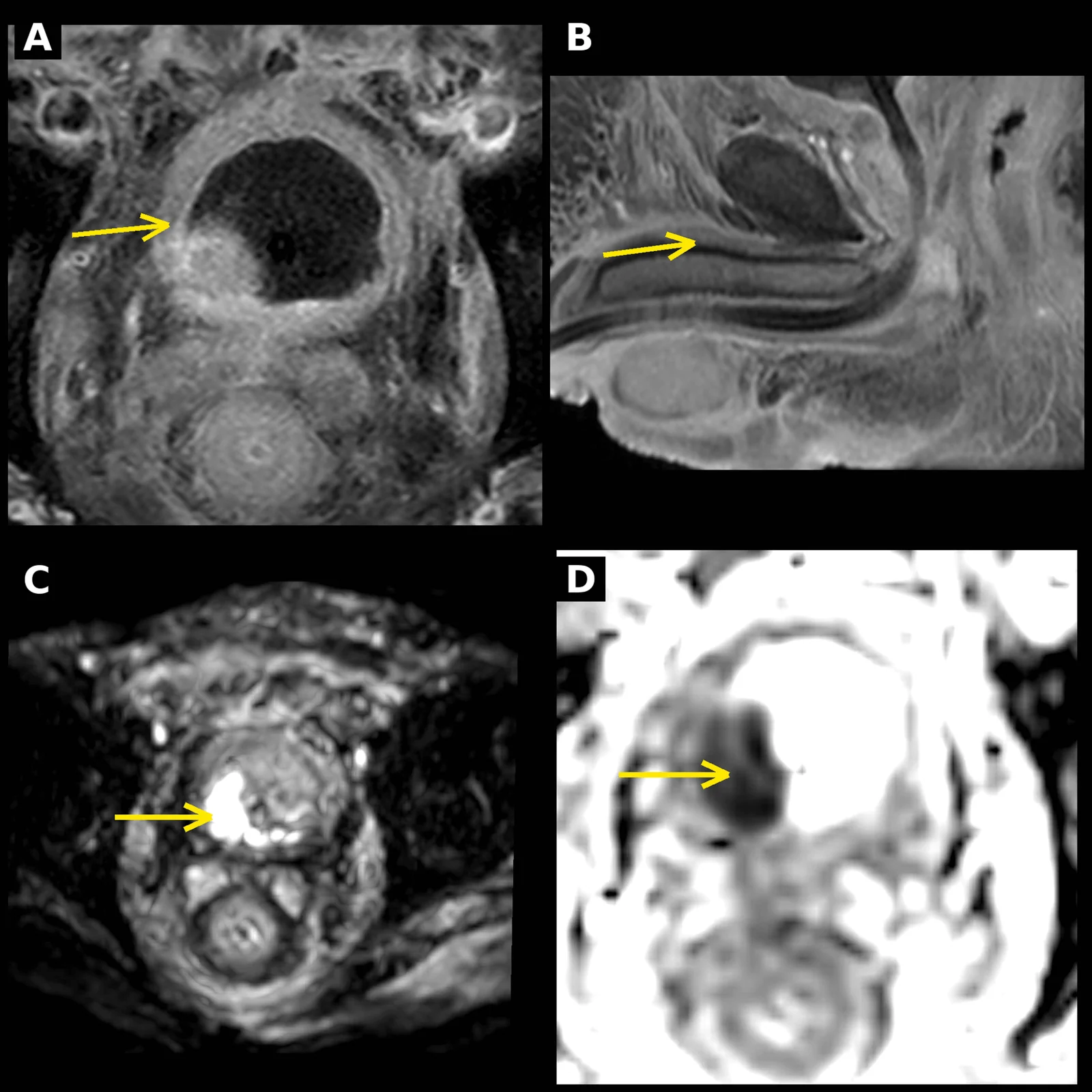
Pelvic magnetic resonance imaging of the bladder lesion. (A) Contrast-enhanced axial image showing irregular, enhancing thickening of the bladder wall corresponding to the tumor (arrow), with the lesion measuring up to 3.7 cm. (B) Sagittal T2-weighted image showing the bladder wall thickening and the extent of the lesion (arrow). (C) Diffusion-weighted image showing focal high signal within the bladder lesion (arrow). (D) Apparent diffusion coefficient map, at the level of the same bladder lesion, showing corresponding low signal (arrow), consistent with restricted diffusion. The diffusion-weighted image and the ADC map are inherently displayed with inverted signal characteristics. The combination of a large lesion with disrupted low-signal muscle layer on T2-weighted imaging marked restricted diffusion, and early enhancement corresponds to VI-RADS category 5, indicating a high likelihood of muscle-invasive disease. VI-RADS: Vesical Imaging-Reporting and Data System, ADC: apparent diffusion coefficient.

Flexible ureteroscopy was performed and directly visualized the bladder lesion as a necrotic area consistent with tumor; biopsies of the bladder and urethral mucosa were obtained from this visualized lesion. A rigid transurethral resection of the bladder tumor was attempted for adequate deep sampling and staging but could not be completed, as the perineal induration prevented advancement of the rigid instrument. During the same procedure, the urology team elected to proceed with an open incisional biopsy of the indurated perineal skin lesion, which was readily palpable and accessible.

Histopathological examination of the bladder and urethral mucosal specimens revealed nonspecific findings consistent with chronic cystitis and urethritis. In contrast, histopathological analysis of the perineal skin specimen demonstrated infiltration of the perineal dermis by invasive urothelial carcinoma, with identifiable mitotic figures. Immunohistochemistry performed on the perineal specimen was positive for GATA-binding protein 3 (GATA3) and cytokeratin 7 (CK7), confirming the urothelial origin of the neoplasm (Figure 2). Thus, the definitive diagnosis was established through the cutaneous perineal biopsy rather than through the conventional transurethral route.

**Figure 2:**
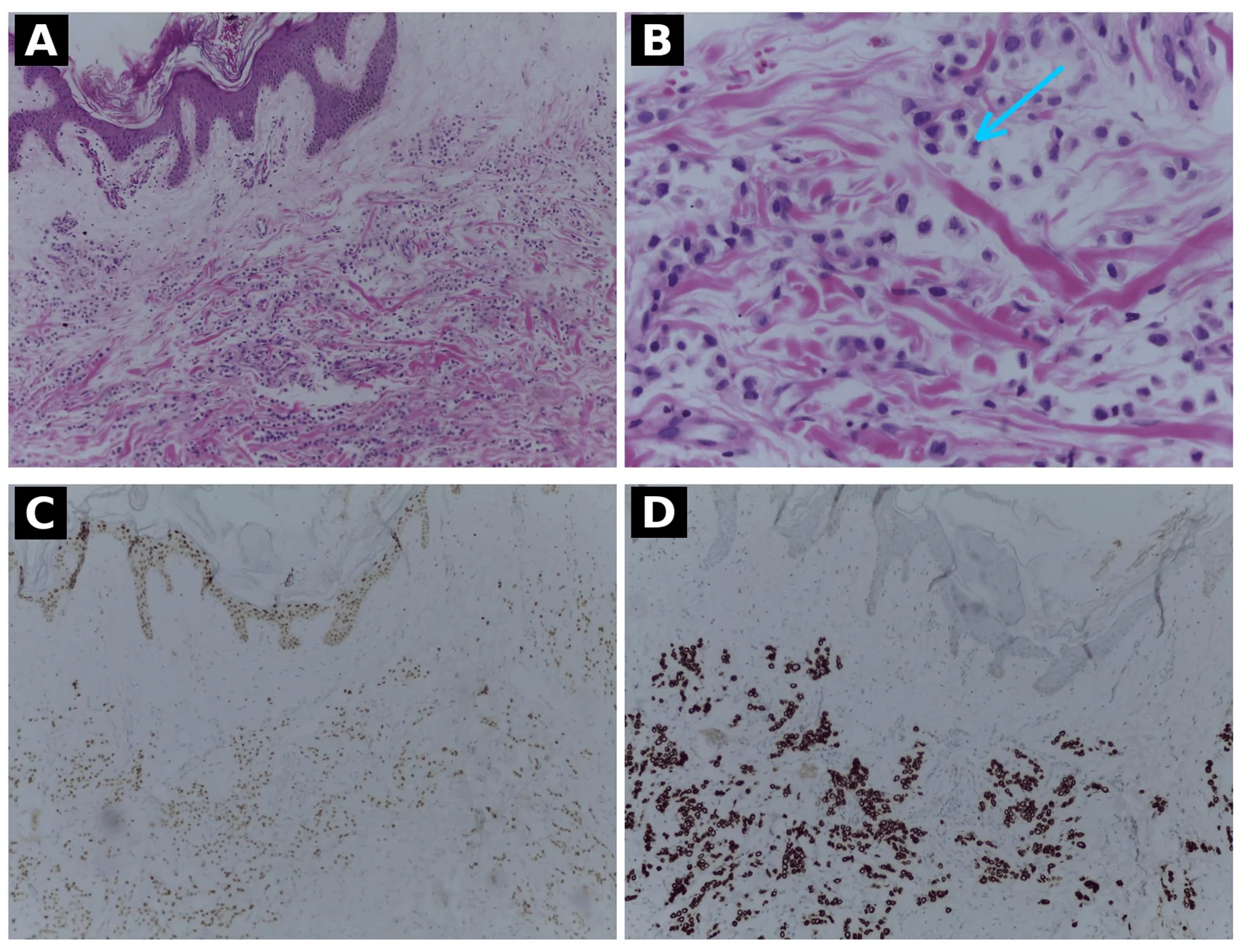
Histopathology and immunohistochemistry of the perineal skin biopsy. (A) Hematoxylin and eosin (H&E), ×100: infiltration of the perineal dermis by urothelial carcinoma. (B) H&E, ×200: neoplastic dermal infiltration with a mitotic figure (arrow). (C) Immunohistochemistry positive for GATA3. (D) Immunohistochemistry positive for CK7. The immunoprofile confirms the urothelial origin of the neoplasm. GATA3: GATA-binding protein 3, CK7: cytokeratin 7.

Staging computed tomography (CT) was subsequently performed, demonstrating the locally advanced pelvic tumor with bladder wall thickening and bilateral upstream urinary tract dilation (Figure 3, complementary to the diagnostic MRI in Figure 1).

**Figure 3:**
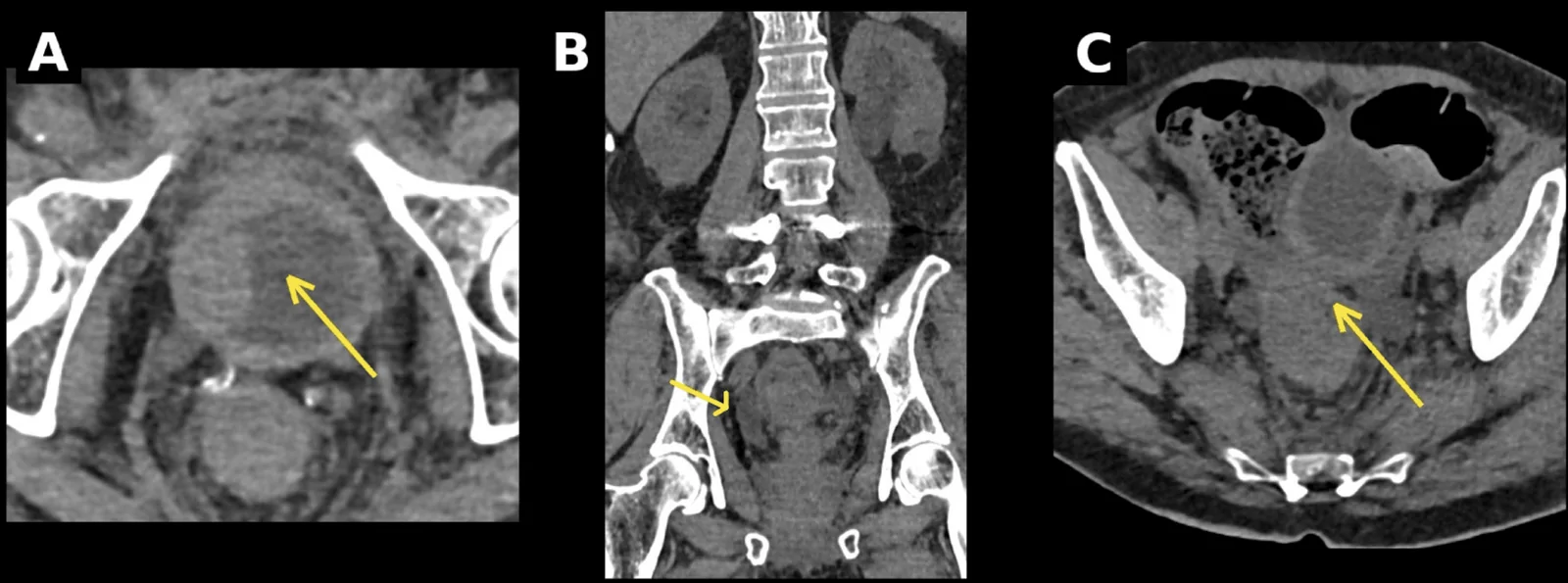
Staging computed tomography, obtained after histological confirmation for locoregional assessment. (A) Axial image showing thickening of the bladder wall by the pelvic tumor (arrow). (B) Coronal image of the pelvis showing the locally advanced tumor with associated ureteral dilation near the bladder (arrow). (C) Lower axial image showing the extent of the pelvic mass (arrow).

Before the first hospital discharge, renal function was preserved, with a serum creatinine of 1.5 mg/dL and adequate urine output; for this reason, the upstream urinary tract dilation seen on the initial imaging was not decompressed at that time. The patient was subsequently discharged. He later returned to the hospital with postrenal acute kidney injury resulting from tumor invasion of the ureteral meatus, which progressed to severe obstructive uropathy with associated urinary tract infection. On readmission, serum creatinine was markedly elevated at 6.02 mg/dL. Bilateral percutaneous nephrostomy and antibiotic therapy were performed in an attempt to relieve the obstruction and control the infection, with improvement of renal function to a serum creatinine of 1.6 mg/dL. Despite these measures, the patient had progressive clinical deterioration and the condition ultimately proved fatal.

## Discussion

The classic presentation of urothelial bladder carcinoma is a man older than 40 years, with a smoking history, presenting with macroscopic hematuria. Early diagnosis allows curative treatment, whereas tumors that are advanced at diagnosis carry a substantially worse prognosis. Beyond tobacco use, which remains the main risk factor, other well-established risk factors for urothelial carcinoma include occupational and environmental exposure to aromatic amines and other industrial chemicals (as encountered in the dye, rubber, leather, and painting industries), chronic exposure to certain agents, and a family history of the disease. In our patient, none of these additional risk factors, including occupational or environmental exposures, were known to be present, and he had no smoking history, which further highlights the atypical nature of this case. Notably, he presented first with lower gastrointestinal complaints, perineal induration, difficulty in defecation, and diarrhea, that initially directed the investigation toward inflammatory bowel disease rather than a urological malignancy. Anatomically, these proctological symptoms can be explained by a locally advanced posteroinferior pelvic tumor exerting mass effect on the rectum and extending into the pelvic floor and perineal region, which links the urological pathology to the initial bowel-related presentation. This atypical presentation illustrates how locally advanced pelvic tumors may mimic colorectal or inflammatory conditions and delay the correct diagnosis.

Diagnosis of urothelial carcinoma is conventionally established through direct biopsy of the suspicious urothelial mucosa via cystoscopy. In the present case, this conventional route yielded only nonspecific inflammatory findings, and it was the biopsy of the indurated perineal skin lesion that proved decisive for establishing the diagnosis. The immunohistochemical profile of the cutaneous specimen, positive for GATA3 and CK7, supported the urothelial origin of the neoplasm, since these markers are characteristically expressed by urothelial carcinoma and help distinguish it from other cutaneous malignancies.

We acknowledge that GATA3 and CK7 positivity, although consistent with urothelial differentiation, is not entirely specific, as both markers can also be expressed in primary cutaneous carcinomas and in extramammary Paget disease, which may occur in the perineal region. A broader confirmatory immunohistochemical panel, including markers such as uroplakin II or III, p63, p40, S100P, thrombomodulin, and CK20, was not performed in this case, which we recognize as a limitation. Nonetheless, the diagnosis of urothelial carcinoma is supported by the overall clinical, radiological, and pathological correlation: a radiologically suspected bladder-centered mass classified as VI-RADS category 5 with contiguity to adjacent pelvic structures, directly visualized as a necrotic tumor on flexible ureteroscopy, together with dermal infiltration by a carcinoma expressing GATA3 and CK7 in an adjacent perineal lesion. Considered together, these findings make urothelial carcinoma with cutaneous involvement the most plausible diagnosis, whether the perineal lesion represents direct extension from the locally advanced pelvic tumor or true cutaneous metastatic spread.

With respect to the biopsy technique, needle biopsy is generally regarded as a less invasive alternative that allows tissue sampling from multiple sites and avoids an incision through tissue involved by malignancy. In the present case, the open incisional biopsy of the perineal lesion reflected an intraoperative decision by the surgical team, taken during the same procedure as the flexible ureteroscopy, and based on the readily palpable and accessible perineal lesion. It is also worth noting that the small working channel of the flexible ureteroscope accommodates only fine biopsy forceps and yields superficial mucosal samples, which may help explain why the transurethral specimens showed only nonspecific chronic inflammation and further supported the decision to biopsy the accessible perineal lesion. This pragmatic approach ultimately provided the diagnostic tissue when the conventional transurethral route was both technically limited and histologically inconclusive.

Cutaneous involvement by urothelial carcinoma, whether by direct extension or metastatic spread, is an uncommon finding, generally seen in advanced disease and associated with a poor prognosis [6,7]. A previously published case report similarly described cutaneous metastasis as the presenting finding of bladder urothelial carcinoma, reinforcing that new skin lesions in patients with a history of, or clinical suspicion for, urothelial malignancy warrant prompt biopsy [6]. This case is notable because the diagnosis was not obtained through the conventional transurethral route but instead through biopsy of a cutaneous perineal lesion, a strategy that may be considered in similar cases in which standard sampling is technically unfeasible or histologically inconclusive.

This report has limitations inherent to a single retrospective case. Deep muscularis propria sampling could not be obtained because rigid transurethral resection was not feasible, and formal pathological tumor staging was therefore not completed. The immunohistochemical characterization was limited to GATA3 and CK7, without a broader confirmatory panel or additional markers to formally exclude other primary sites, and it was not possible to definitively distinguish direct contiguous extension from true cutaneous metastasis. Despite these constraints, the combined clinical, radiological, and pathological findings support the reported diagnosis and illustrate a decisive alternative diagnostic route when conventional sampling is unfeasible or inconclusive.

## Conclusions

Bladder urothelial carcinoma should remain a diagnostic consideration in patients presenting with perineal induration and lower urinary or gastrointestinal symptoms, even in the absence of classic risk factors such as smoking. When conventional transurethral biopsy is technically difficult or yields nonspecific results, biopsy of an associated cutaneous lesion, supported by immunohistochemistry, can provide a decisive diagnostic alternative. Awareness of this atypical presentation may help clinicians reach a timely diagnosis in comparable cases.
